# Comparative study on the nocturnal activity of phlebotomine sand flies in a highland and lowland foci of visceral leishmaniasis in north-western Ethiopia with special reference to *Phlebotomus orientalis*

**DOI:** 10.1186/s13071-017-2339-6

**Published:** 2017-08-23

**Authors:** Esayas Aklilu, Araya Gebresilassie, Solomon Yared, Mizan Kindu, Habte Tekie, Meshesha Balkew, Alon Warburg, Asrat Hailu, Teshome Gebre-Michael

**Affiliations:** 1 Department of Biology, Mada Walabu University, Bale-Robe, Ethiopia; 2 Department of Biology, Jigjiga University, Jigjiga, Ethiopia; 3 Department of Microbiology and Parasitology, Mada Walabu University, Bale-Robe, Ethiopia; 4 Department of Zoological Sciences, Addis Ababa University, Addis Ababa, Ethiopia; 5 Aklilu Lemma Institutes of Pathobiology, Addis Ababa University, Addis Ababa, Ethiopia; 6 Faculty of Medicine, The Hebrew University, Hadassah Medical School, Jerusalem, Israel; 7 Department of Microbiology, Immunology and Parasitology, College of Health Sciences, Addis Ababa University, Addis Ababa, Ethiopia

**Keywords:** *Phlebotomus orientalis*, Nocturnal activity, Visceral leishmaniasis, Highland, Lowland, North-western Ethiopia

## Abstract

**Background:**

*Leishmania donovani*, the causative agent of visceral leishmaniasis (VL), is most probably vectored by *Phlebotomus orientalis* in north-western Ethiopia. The aim of this study was to determine and compare the nocturnal activity patterns of *Ph. orientalis* in VL endemic foci of Libo-Kemkem (highland) and Metema (lowland) districts of north-western Ethiopia.

**Methods:**

Sampling of sand flies was conducted bimonthly from January-May 2012 in the highland and from March-June 2013 in the lowland. Sand flies were sampled using two CDC light traps placed in compounds occupied by both cattle and humans. Sampling of sand flies started at 18:00 h and ended at 06:00 h. Every hour, a cage was replaced by another cage.

**Results:**

In total, 9479 nocturnally active sand flies were collected from both study areas. Six *Phlebotomus* species (*Ph. orientalis*, *Ph. duboscqi*, *Ph. papatasi*, *Ph. bergeroti*, *Ph. rodhaini* and *Ph. martini*) and several *Sergentomyia* spp. were identified. In both areas, of the six *Phlebotomus* spp., *Ph. orientalis* was the preponderate. In the highland, the hourly activity pattern of *Ph. orientalis* females was higher before midnight with a peak in density between 22:00–23:00 h, whereas in the lowland after midnight between 03:00–04:00 h.

**Conclusions:**

The present study showed that *Ph. orientalis* females exhibited different nocturnal activity patterns with a peak in the early part of the night in the highland and in the latter part of the night in the lowland areas. As the risk of acquiring *L. donovani* infections vary in the two areas, appropriate control strategies should be developed according to the activity of *Ph. orientalis* in the respective areas.

## Background

Phlebotomine sand flies (Diptera: Psychodidae) are small biting insects of considerable public health importance in many parts of the world, where they are the vectors of human pathogens. Most importantly, they transmit etiological agents of leishmaniasis [1]. Leishmaniases are a group of diseases caused by protozoan parasites of the genus *Leishmania*. The diseases manifest from self-healing cutaneous leishmaniasis (CL) to life-threatening visceral leishmaniasis (VL) [2]. In the Old World, the implicated vectors of the disease belong to the genus *Phlebotomus* [3].

*Leishmania donovani*, the causative agent of VL, is transmitted mainly by two sand fly species in east Africa, *Phlebotomus martini* and *Ph. orientalis*. *Phlebotomus martini* is frequently associated with termite mounds in Kenya and southern Ethiopia whereas *Ph. orientalis* is associated with *Acacia-Balanites* forests and black cotton soil in Sudan, South Sudan, north and north-western Ethiopia [4–8].

In many parts of the Old World, sand flies of the genus *Sergentomyia* are the most abundant phlebotomine species [9–12]. Species of this genus are known to transmit reptile leishmaniasis [13, 14]. However, there are contradictory reports regarding about the potential role of the *Sergentomyia* species as vectors of human leishmaniasis. Kanjanopas et al. [15] detected DNA of *Leishmania siamensis* (the causative agent of VL in Thailand) using molecular methods in *Se. gemmea*. Similarly, DNA of *L. major* was detected in *Se. minuta* in Portugal and Tunisa [16, 17]. More recently, Senghor et al. [18] isolated *L. infantum*, the causative agent of canine leishmaniasis in Senegal, from four females (two *Se. dubia* and two *Se. schwetzi*) using dissection. Conversely, experimental study on Ethiopian *Se*. *schwetzi* indicated that this species does not support the development of the three *Leishmania* spp. (*L. donovani, L. infantum* and *L.major*) [19].

Most sand fly species are either crepuscular, with peaks of activity soon after sunset and before dawn, or nocturnal [20, 21]. Nocturnal activity of sand flies consists of a number of discrete components, predominantly search for blood meals, sugar meals, mates and breeding sites [22]. Such behavior of sand flies is governed by a range of factors such as internal biological clock, daily changes in light intensity and other abiotic factors, including temperature, relative humidity, rain, and wind speed [23].

Various studies have been reported on nocturnal activity patterns of *Ph. orientalis* in north-western and northern Ethiopia. Ashford et al. [24] reported that a peak activity of *Ph. orientalis* reached shortly after sunset in Arbaya after which the activity of the sand flies subsided concomitantly as the temperature dropped from 16 °C. Yared et al. [25] indicated that *Ph. orientalis* was active throughout the night and reached a peak between 01:00–03:00 h in Kafta-Humera, north-western Ethiopia. Similarly, in northern Ethiopia Gebresilassie et al. [26] showed that a peak nocturnal activity of this species was before midnight (22:00–23:00 h).

Adequate knowledge of the nocturnal activity pattern of sand flies in general, and vector species in particular, would contribute to the understanding of the epidemiology of sand fly-borne diseases, as it indicates the time when a person is most likely to be bitten and get the diseases [27, 28]. However, such information is absent in Libo-Kemkem and Metema districts, where kala-azar is endemic and causes major problem in the two districts. Therefore, the present study was designed to determine and compare such behavior of *Ph. orientalis* in the two ecologically distinct foci of VL in the region. In addition, an attempt was also made to elucidate the effect of local weather such as temperature and relative humidity on the nocturnal activity of the species in both foci.

## Methods

### Study areas

#### Libo-Kemkem District

An entomological study was carried out in the village of Bura in Libo-Kemkem District (12°04′N, 37°45′E) in the Amhara Regional State, north-western Ethiopia. The district is found about 645 km northwest of Addis Ababa. The district is a highland area with an average altitude of 2000 m above sea level (masl). The mean annual temperature of the area is 20.3 °C. It has uni-modal type of rain, with annual total rainfall of 1350 mm. The natural vegetation coverage of the area (mostly *Acacia seyal*) has been immensely reduced mainly for agricultural purpose, construction of houses and fire wood. Agriculture and allied activities are the most important source of subsistence for the majority of the population. They principally produce *teff*, maize, millet, bean, sunflower, rice and cotton during the main rainy season. They also raise a large number of livestock, including cattle, sheep, goats and poultry.

#### Metema District

Nocturnal activity of *Ph. orientalis* also conducted in the village of Kokit in Metema District (12°46′N, 36°24′E) in the Amhara Regional State, north-western Ethiopia. The site is located 860 km northwest of Addis Ababa and 180 km southwest of Gondar town. The district is a lowland plain with an average altitude of 750 masl. The district has uni-modal rainfall, with annual rainfall ranging between 850 and 1000 mm. The rain starts in June and extends until the end of September. Unlike Libo-Kemkem, rainfall in Metema can be erratic. The dry season of the area starts in October and extends until the end of May. During this period, the temperature usually ranges between 27 °C and 32 °C and the daily maximum temperature reaches 41 °C in April. The characteristic vegetation of the area is wooded savannah which is alternating with grasslands. The main trees are *A. syeal*, *Balanites aegyptiaca*, and *Zyzyphus spina-christa*. People in the area depend on agricultural activities as main source of income. Farming activities include cultivation of cash crops (such as sesame and cotton), and staple crops (*teff*, maize and sorghum). They also raise cattle, sheep and goats.

### Sand fly sampling

The nocturnal activity of sand flies was determined using two Centers for Disease Control and Prevention (CDC) light traps placed in compounds shared by both cattle and humans, so as to adequately sample the host-seeking sand flies. Sampling of sand flies was conducted bimonthly from January-May 2012 in the highland and from March-June 2013 in the lowland. These periods were the active seasons of sand fly activity in the two study areas. Collection of sand fly specimens using CDC light traps was started at 18:00 h and ended at 06:00 h. Every hour, a collection cage was replaced by another cage labelled with date and time of collection.

### Dissection of sand flies for age determination

The morning after collection, the hourly catches of *Ph. orientalis* females were sorted out according to their abdominal status (unfed, blood-fed and gravid/semi-gravid) under the dissecting microscope. Dissection was performed on unfed females to separate into parous and nulliparous as described by Gebre-Michael et al. [29]. Male *Phlebotomus* spp. and both sexes of *Sergentomyia* spp. were preserved in 70% ethanol for species identification.

### Mounting and identification of sand flies

Sand flies collected during the study periods were mounted on microscope slides in Hoyer’s medium with their heads separate from thoraces and abdomens. Identification of the species was made by examining male genitalia, and the female spermathecae and pharynx according to the morphological keys of Quate [30] and Abonnenc & Minter [31]. Additional morphological keys of Lane & Fritz [32] and Gebre-Michael & Medhin [33] were also used to separate sympatric species of the subgenus *Phlebotomus* in Metema.

### Recording weather variables

Hourly weather variables (temperature and relative humidity) were recorded from dusk to dawn using data logger (HOBO Microstation, Massachusetts, USA) during the collection nights.

### Data analysis

The mean densities of sand flies were computed as numbers of flies per light trap per h. Prior to analysis of the data, normality of the data was checked using Shapiro-Wilk test. Student’s t-test was used to compare the activity of females and males of *Ph. orientalis* before and after midnight. Kruskal-Wallis test was used to compare the hourly activity of males, females and total *Ph. orientalis* populations and proportion of parous rates. Correlation between hourly recorded weather variables (temperature and relative humidity) and density of *Ph. orientalis* was assessed using Spearman’s rank correlation coefficient.

## Results

### Species composition and relative abundance of nocturnally active sand flies

Table 1 shows nocturnally active sand fly species collected during the study periods in both study areas. Overall, 9479 sand flies were collected. In Libo-Kemkem, a total of 3438 (1981 males and 1457 females) sand flies were collected from January-May, 2012 which consisted one *Phlebotomus* species (*Ph. orientalis*) and five *Sergentomyia* spp. (*Se. clydei*, *Se. squamipleuris*, *Se. bedfordi*, *Se. schwetzi* and *Se. africana*). Of these species, *Ph. orientalis* was the most preponderate species and accounted 96.54% of the total collection (Table 1). In Metema, a total of 6041 (2193 males and 3848 females) sand flies were caught from March-June 2013, which included six species of *Phlebotomus* (*Ph. orientalis*, *Ph. duboscqi*, *Ph. papatasi*, *Ph. bergeroti*, *Ph. rodhaini* and *Ph. martini*) and seven species of *Sergentomyia* (*Se.clydei*, *Se. squamipleuris*, *Se. bedfordi*, *Se.schwetzi*, *Se. africana*, *Se. adleri* and *Se.antennata*). In the lowland, the six most prevalent species in descending order were *Se. clydei* (40%), *Se. bedfordi* (21.9%), *Ph. orientalis* (13.5%), *Se. schwetzi* (9.0%), *Se. africana* (6.2%) and *Se. squamipleuris* (5.7%).

**Table 1:** Nocturnally active sand fly species captured using CDC light trap in Libo-Kemkem District (January-May 2012) and Metema District (March-June 2013)

Species	Libo-Kemkem District			Metema District			Overall		
	Male	Female	Total (%)	Male	Female	Total (%)	Male	Female	Total (%)
*Phlebotomus orientalis*	1943	1376	3319 (96.54)	442	371	813 (13.5)	2385	1747	4132 (43.59)
*Ph. duboscqi*	–	–	–	2	2	4 (0.07)	2	2	4 (0.04)
*Ph. papatasi*	–	–	–	1	5	6 (0.10)	1	5	6 (0.06)
*Ph. bergeroti*	–	–	–	7	8	15 (0.30)	7	8	15 (0.16)
*Ph .*r*odhaini*	–	–	–	4	9	13 (0.20)	4	9	13 (0.14)
*Ph. martini*	–	–	–	1	0	1 (0.01)	1	0	1 (0.01)
*Sergentomyia clydei*	0	4	4 (0.12)	801	1619	2420 (40.00)	801	1623	2424 (25.57)
*Se. squamipleuris*	31	52	83 (2.41)	84	258	342 (5.70)	115	310	425 (4.48)
*Se. bedfordi*	7	16	23 (0.67)	395	927	1322 (21.90)	402	943	1345 (14.18)
*Se. schwetzi*	0	2	2 (0.06)	139	408	547 (9.00)	139	410	549 (5.80)
*Se. africana*	0	7	7 (0.20)	221	152	373 (6.20)	221	159	380 (4.00)
*Se. adleri*	–	–	–	0	7	7 (0.12)	0	7	7 (0.10)
*Se. antennata*	–	–	–	96	82	178 (2.90)	96	82	178 (1.87)
Total	1981	1457	3438 (36.30)	2193	3848	6041 (63.70)	4174	5305	9479

### Nocturnal activity pattern of *Ph. orientalis*

#### Libo-Kemkem District

**Fig. 1:**
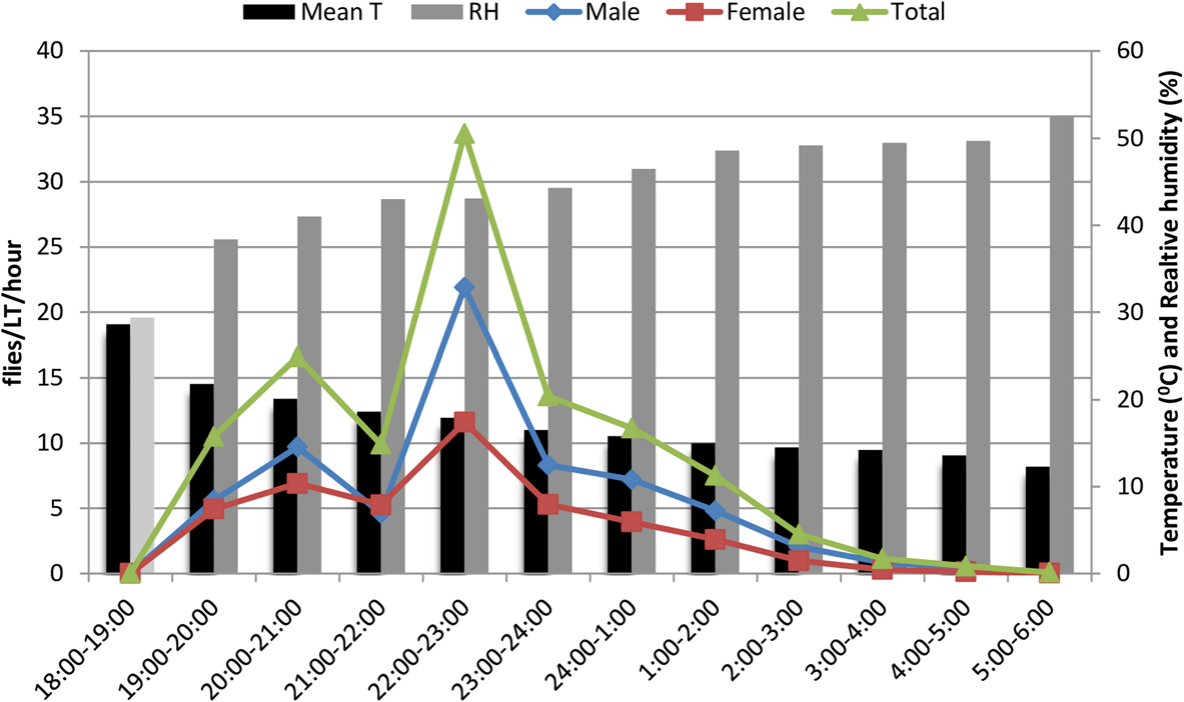
Nocturnal activity pattern of *Ph. orientalis* and mean hourly temperature and relative humidity in Libo-Kemkem District (January-May 2012)

During each collection night, the male to female ratio varied with the hour of collection. The overall sex ratio and range were 1.4 and 0.9–3.4, respectively. The hourly activity pattern of *Ph. orientalis* was significantly different for females (Kruskal-Wallis test, *χ* ^2^ = 31.9, *df* = 11, *P* < 0.05), males (*χ* ^2^ = 21.7, *df* = 11, *P* < 0.05) and both sexes of the species (*χ* ^2^ = 28.1, *df* = 11, *P* < 0.05). Increased density of flies for both sexes were observed between 21:00–24:00 h, with a peak between 22:00–23:00 h (Fig.1). When the density of females grouped before and after midnight, a significant difference was observed in the two periods (*F*_(1, 10)_ = 0.974, *P* = 0.026), since more females were caught before midnight. However, such difference was absent on the activity of males and both sexes (*P* > 0.05). After midnight, the densities of both sexes subsided steadily throughout the night. During the peak activity hours, the mean temperature and relative humidity were 17.9 ± 2.43 °C and 43.1 ± 6.2%, respectively (Fig. 1).

**Fig. 2:**
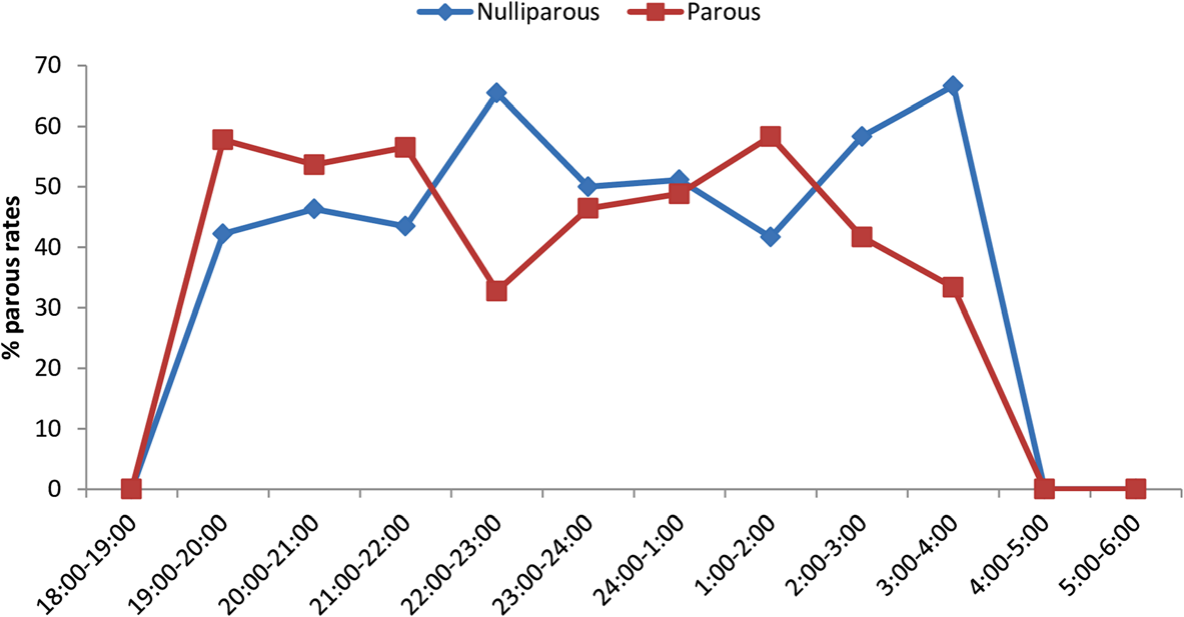
Hourly proportions of parous and nulliparous females *Ph. orientalis* in Libo-Kemkem District (January-May 2012)

Of 285 unfed females *Ph. orientalis* dissected for parity, 48.8% (*n* = 139) were found to be parous. There was a significant difference in the hourly proportion of parous females (Kruskal-Wallis test, *χ* ^2^ = 21.4, *df* = 11, *P* = 0.029), in which the majority of parous sand flies were caught before midnight (105/139, 75.5%). In general, parous females have two major peaks of activity during the night, one before midnight (19:00–22:00 h) and another after midnight (01:00–02:00 h). Similar bimodal peaks in activity for nulliparous females were also observed, the first peak was between 22:00–23:00 h and the second peak was between 03:00–04:00 h (Fig. 2).

#### Metema District

**Fig. 3:**
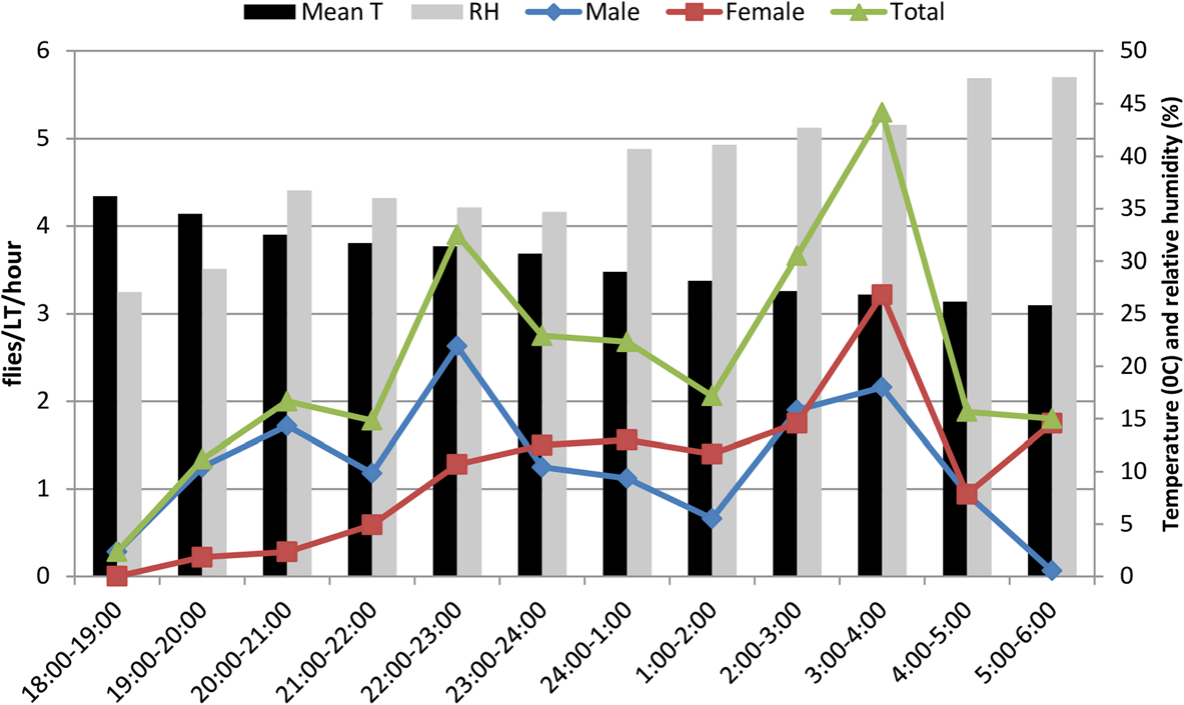
Nocturnal activity pattern of *Ph. orientalis* and hourly mean temperature and relative humidity in Metema District (March-June 2013)

During each collection night, the male to female ratio varied with the hour of collection. The overall ratio and range were 1.2 and 0.04–9.00, respectively. Hourly activity pattern of *Ph. orientalis* in relation to mean hourly temperature and relative humidity is depicted in Fig. 3. The activity of males of *Ph. orientalis* started after sunset and continued throughout the night (*χ* ^2^ = 17.6, *df* = 11, *P* > 0.05) with slight peak between 22:00–23:00 h. Like the activity of males, the activity of total *Ph. orientalis* population was also continuous (*P* > 0.05). Males were predominating over females until their peak activity (22:00–23:00 h) (2.6 ± 1.0/LT/h). The activity of females began one hour later than males, with peak was observed between 03:00–04:00 h(3.21 ± 1.4/LT/h). Unlike males, the hourly activity pattern of *Ph. orientalis* females was not continuous (*χ* ^2^ = 21.06, *df* = 11, *P* < 0.05). The activity of *Ph. orientalis* females continued until 06:00 h, but the activity of males decreased drastically after 05:00 h. When the density of females grouped before and after midnight, there was a significant difference in the activity of females before and after midnight (*F*_(1, 10)_ = 0.007, *df* = 10, *P* = 0.02), as more females were caught after midnight. However, such difference was not observed on the activity of male sand flies and both sexes of *Ph. orientalis* (*P* > 0.05). During the peak activity hours, the mean temperature and relative humidity were, 26.85 ± 0.28 °C and 43 ± 8.5 (%), respectively (Fig. 3).

An attempt was also made to dissect unfed females *Ph. orientalis* to determine hourly parity rate. However, due to unfavorable (hot) weather conditions, only a few (*n* = 33) unfed females *Ph. orientalis* were dissected. Out of these, 21 (64%) of them were parous. Due to the low number of dissected flies, determination of hourly parous rate was not possible.

### Nocturnal activity pattern of *Sergentomyia* species

**Fig. 4:**
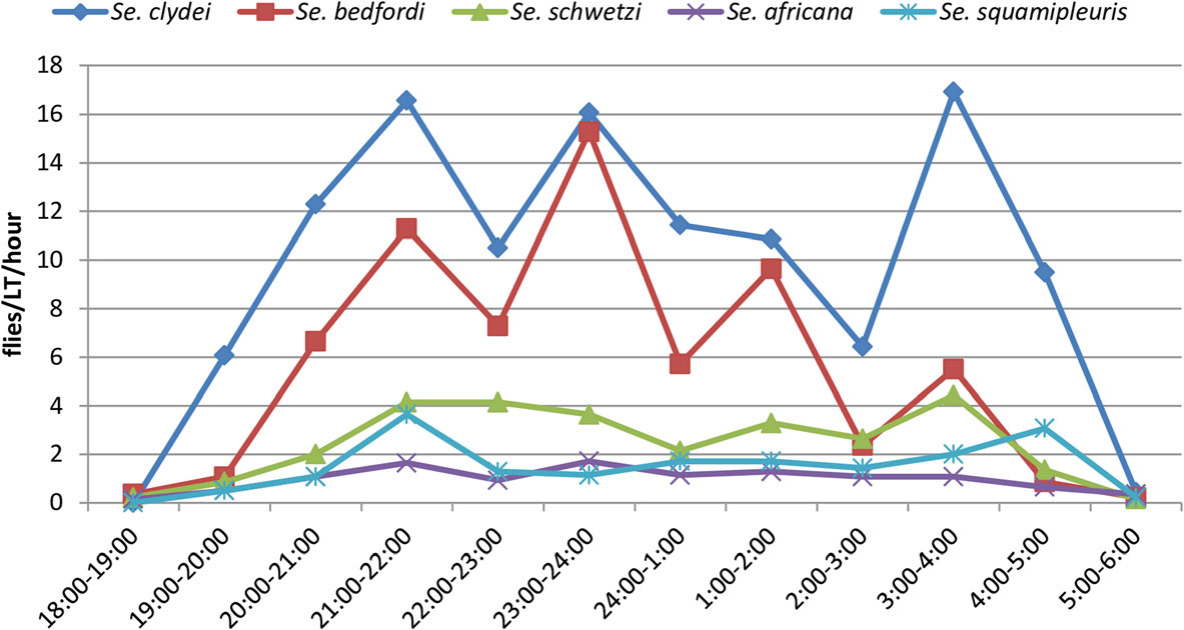
Nocturnal activity pattern of female *Sergentomyia* species collected from Metema District (March-June 2013)

In Libo-Kemkem District, *Sergentomyia* species were not sufficiently abundant for their nocturnal behavior to be studied. However, in Metema District, activity patterns of five female *Sergentomyia* species (*Se.clydei*, *Se. bedfordi*, *Se. schwetzi*, *Se. africana* and *Se. squamipleuris*) were analyzed and presented in Fig. 4. In general females of these species showed two to four peaks in activity patterns. For instance, females of *Se. squamipleuris* had two peaks. The first peak was before midnight between 21:00–22:00 h and the second peak was after midnight between 04:00–05:00 h. Females of *Se. clydei* showed three peaks in activity patterns. The first and the second peaks were before midnight from 21:00–22:00 h and 23:00–24:00 h, respectively, whereas the last peak was after midnight between 03:00–04:00 h. Like females of *Se. clydei*, females of *Se. schwetzi* had also three peaks in activity patterns. Of the three peaks, the major one was observed after midnight between 03:00–04:00 h. Females of *Se. bedfordi* showed four peaks in activity patterns with the major one was between 23:00–24:00 h (Fig. 4).

### Effect of weather variables on nocturnal activity of *Ph. orientalis*

The Spearman correlation coefficient analysis between nocturnal activity pattern of *Ph. orientalis* and hourly mean temperature and relative humidity of both districts is depicted in Table 2. In Libo-Kemkem District, hourly mean temperature had significant positive correlation with mean hourly activity of male, female and total population of *Ph. orientalis* (*P* < 0.05). Unlike temperature, relative humidity had negative association with male, female and total population of *Ph. orientalis* though not statistically significant (*P* < 0.05). Unlike in Libo-Kemkem, in Metema District, the hourly mean temperature had significant negative association (*P* = 0.036) with the mean hourly activity of female *Ph. orientalis*, but statistically insignificant negative association with the mean hourly activity of male and total *Ph. orientalis*. Unlike temperature, the relative humidity had positive and statistically significant association with mean hourly activity of female and combined population of *Ph. orientalis* (*P* < 0.05).

**Table 2:** Correlation of hourly mean temperature and relative humidity with mean hourly activity of *Ph. orientalis* in Libo-Kemkem and Metema districts

Species	Libo-Kemkem District				Metema District			
	Mean temperature		Relative humidity		Mean temperature		Relative humidity	
	ρ^c^	*P*-value	ρ^c^	*P*-value	ρ^c^	*P*-value	ρ^c^	*P*-value
Male	0.389	0.002^b^	-0.24	0.068	-0.091	0.54	0.167	0.256
Female	0.45	< 0.0001^b^	-0.252	0.052	-0.304	0.036^a^	0.410	0.004^b^
Total	0.44	< 0.0001^b^	-0.234	0.07	-0.199	0.174	0.293	0.043^a^

^a^Correlation is significant at the 0.05 level

^b^Correlation is significant at the 0.01 level

^c^ρ, Spearman’s correlation coefficient

### Monthly variation in nocturnal activity pattern of *Ph. orientalis*

**Fig. 5:**
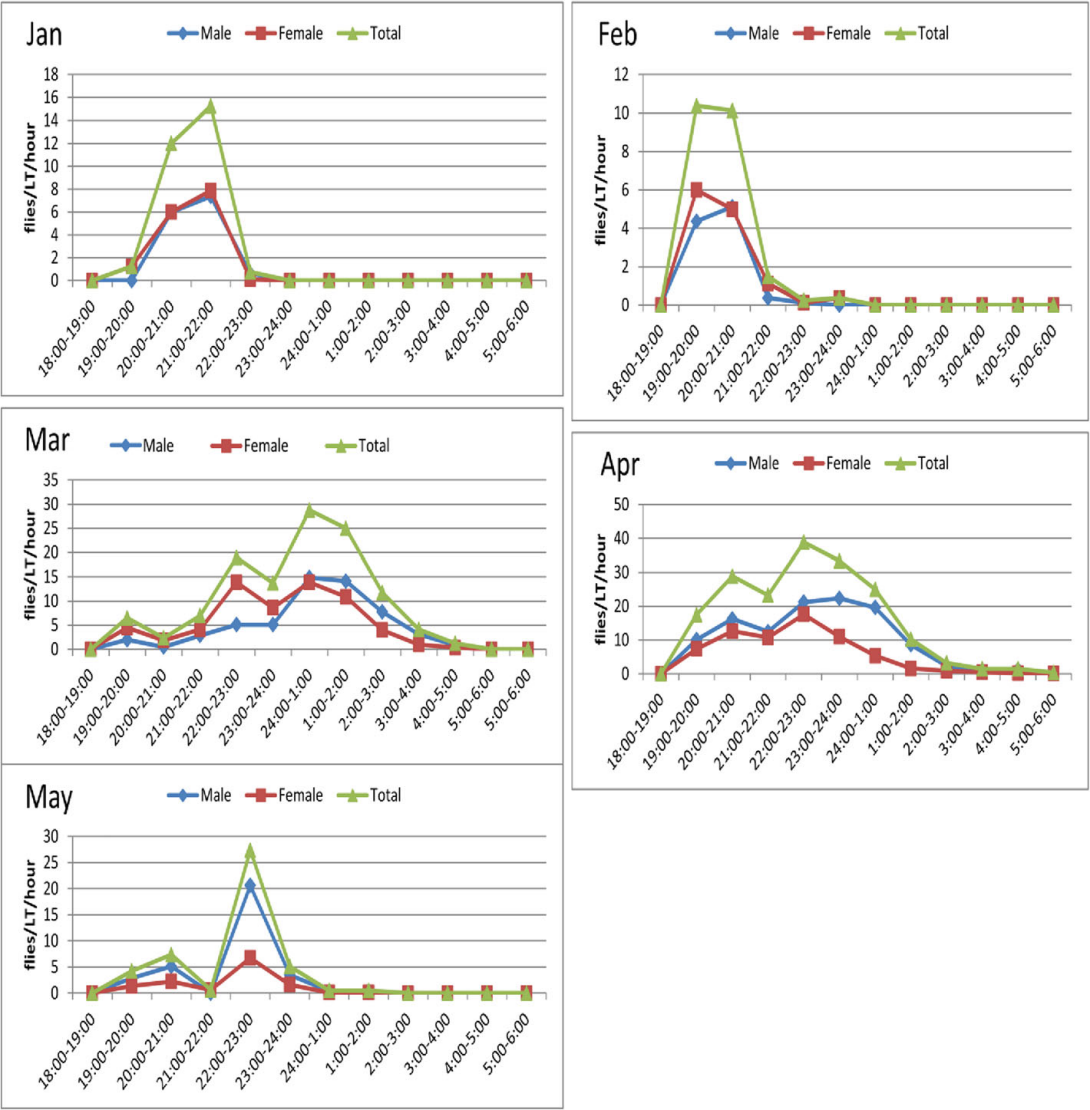
Monthly variation of nocturnal activity of *Ph. orientalis* in Libo-Kemkem District (January-May 2012)

**Fig. 6:**
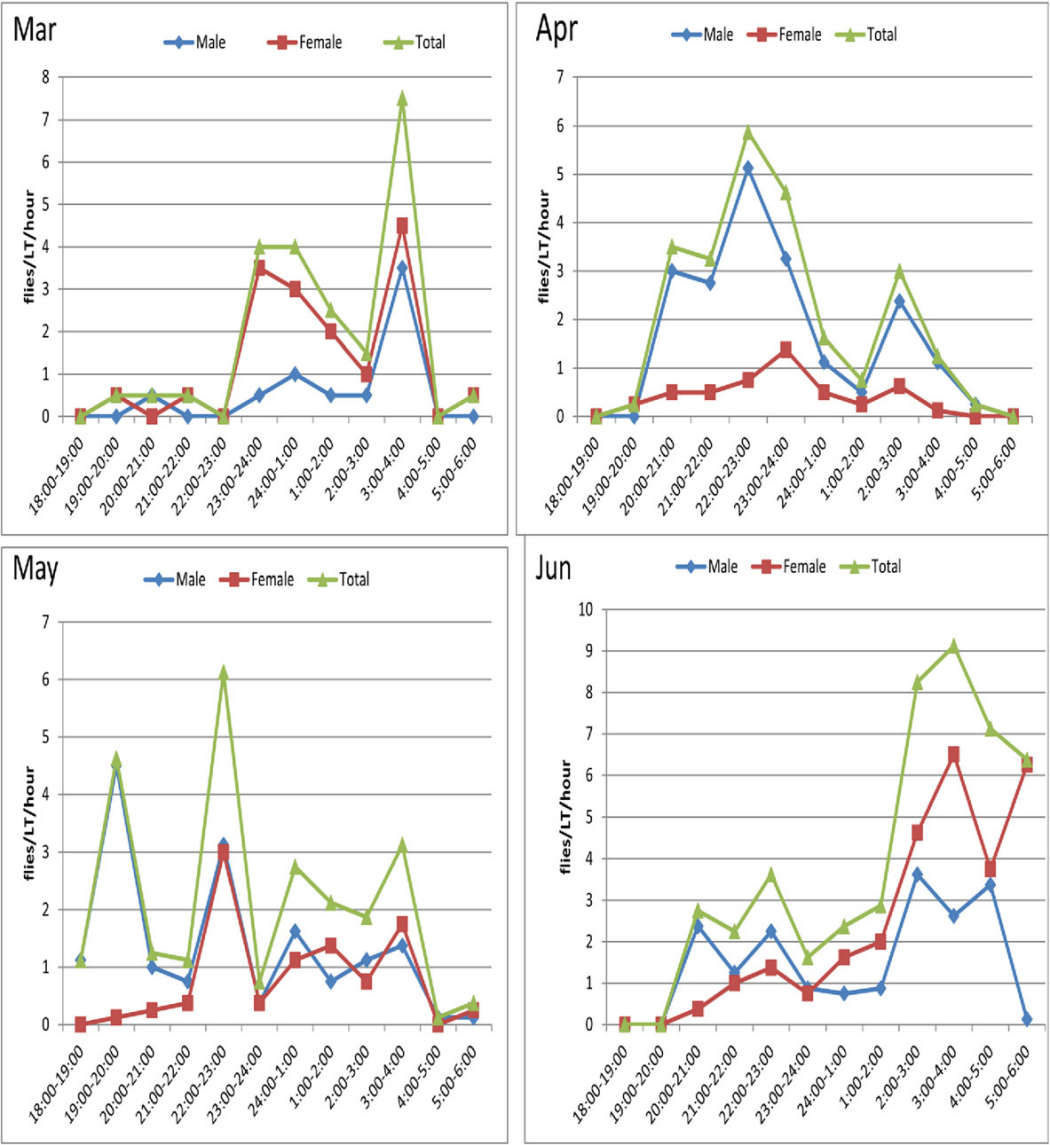
Monthly variation of the nocturnal activity of *Ph. orientalis* in Metema District (March-June 2013)

In Libo-Kemkem District, peak nocturnal activity of *Ph. orientalis* showed monthly variation (Fig. 5). In January and February, the peak periods of activity were about early evening between 19:00–22:00 h, whereas March-May the peak periods of activity were generally around midnight (22:00–01:00 h). Like the population of Libo-Kemkem, the peak nocturnal activity pattern of the Metema population also showed monthly variation (Fig. 6). In March and June, the peak activity pattern was latter in the night between 03:00–04:00 h, whereas in April and May were between 22:00–23:00 h.

## Discussion

The activity pattern of phlebotomine sand flies is either crepuscular or nocturnal [1, 20]; however, such behavioral pattern greatly shows variation between different species in the same locality or within the same species in different localities. In the present study, the hourly activity pattern of highland and lowland populations of *Ph. orientalis* were investigated. The activity of *Ph. orientalis* in the highland area was mainly restricted in the first half of the night, with a peak between 22:00–23:00 h. After midnight the density of this species started to subside drastically. In contrast, *Ph. orientalis* in the lowland area was active throughout the night (from dusk to dawn), with a peak activity in the latter hour of the night between 03:00–04:00 h. Such difference in peak activity periods between the two populations may be due to highly contrasting weather and ecological variations between the two study areas. Moreover, the sampling periods (months and years) in the two geographical areas were different. Previous studies on a peak nocturnal activity of the species reported contrasting results. In Sudan, Quate [30] found a large number of *Ph. orientalis* between 21:30–22:00 h using human biting collection. In the same country, using the same collection method, Schorscher & Goris [34] reported that biting activity of *Ph. orientalis* occurred throughout the night until sunrise. In Ethiopia, Ashford et al. [24] indicated the peak biting activity of *Ph. orientalis* started shortly after sunset in the highland area of Belessa. In Kafta-Humera (north-western Ethiopia), Lemma et al. [35] reported the peak activity period of this species to be between 24:00–01:00 h around an animal shelter. In Sheraro (northern Ethiopia), Gebresilassie et al. [26] reported similar activity pattern like Metema population, where most females of *Ph. orientalis* were active after midnight with a peak density between 24:00–03:00 h.

The difference in nocturnal activity pattern of *Ph. orientalis* in the highland and lowland areas has its own epidemiological implications in the respective areas. In the highland area, the local population is at great risk of acquiring VL before midnight from the bite of the flies. Unlike the highlanders, people in the lowland area are at risk of being infected with *L. donovani* by the bite of *Ph. orientalis* throughout the night, as the majority of the people sleep outdoors for various reasons, such as to keep their cattle from theft and to work on the farms. This behavior is not different to the observations made in the Kafta Humera and Sheraro lowlands in north-western and northern Ethiopia, respectively [25, 26, 35].

In Metema District, nocturnal activity patterns of females of five *Sergentomyia* species (*Se. clydei*, *Se. bedfordi*, *Se. schwetzi*, *Se. africana* and *Se. squamipleuris*) were also analyzed. Of these, *Se. clydei* was the predominate species and exhibited three peaks in activity patterns. The two peaks were observed before midnight between 21:00–24:00 h and the last peak was after midnight between 03:00–04:00 h. A previous study in Oman indicated that females of this species were active during the first half of the night and showed a single peak at 22:00 h, which coincides with one of the peaks of the present study [36].

In the current study, the peak nocturnal activity periods of *Ph. orientalis* in both study areas showed monthly variation. In the highland area, for instance, the peak activity hours of the species shifted from early evening 19:00–22:00 h in January to 24:00–01:00 h in March. In the same manner, in the lowland area the peak activity of this species shifted from 03:00–04:00 h in March to 22:00–23:00 h in May. Such variation might be due to changes in weather variables (such as temperature and relative humidity) between the months. Similarly, in Iraq Coleman et al. [37] observed a shift in peak activity periods of sand flies (*Ph. alexandri*, *Ph. papatasi*, *Ph. sergenti* and *Sergentomyia* spp.) from early in the evening in April and October to later in the night in May and June. Also, from Morocco Guernaoui et al. [28] reported difference in the activity of *Ph. sergenti* between August (markedly crepuscular) and October (peak in activity between 20:00–22:00 h).

The correlation analysis between hourly activity patterns of *Ph. orientalis* and hourly mean temperature and relative humidity showed contrasting results between the highland and lowland areas. In the highland, mean temperature had positive and statistically significant association with the density of *Ph. orientalis*, whereas the relative humidity had negative correlation. Likewise, Ashford et al. [24] noted similar association between temperature and biting activity of this species in another highland area, Belessa. Ashford et al. [24] noticed that the activity of the species was considerably reduced when the temperature was below 16 °C, which is comparable to the present study. As the temperature dropped from 17.9 ± 2.4 °C (the mean temperature at the time of peak hourly activity of *Ph. orientalis*), the activity of the species concomitantly started to reduce. In Morocco, Guernaoui et al. [28] reported the direct association between temperature and hourly abundance of *Ph. sergenti* and *Ph. perniciosus*. In the highland area, temperature appears to be the determining factor for the nocturnal activity of *Ph. orientalis*, although the role of other meteorological factors such as wind speed cannot be ruled out.

In the lowland however, mean temperature and relative humidity had negative and positive relationships with the hourly activity pattern of the species, respectively. In this area, the most important factor which affects the nocturnal activity of *Ph. orientalis* seems to be relative humidity. The importance of high relative humidity for sand flies activity was reviewed by Lewis &Ward [38]. In Kafta Humera, in north-western Ethiopia, hourly activity pattern of *Ph. orientalis* also had similar relationship with relative humidity [25]. Likewise, Dinesh et al. [39] pointed out the importance of high relative humidity for blood-feeding habits of *Ph. argentipes* in India. Contrary to the present observation and previous reports, Roberts [40] revealed that nocturnal activity of *Ph. alexandri* was greatest on the nights with low humidity in Oman.

In the lowland area, females and males of *Ph. orientalis* did not have similar periodicities like the highland population. The hourly activity pattern of males commenced 1 hour earlier than females which apparently related to mating behavior of *Ph. orientalis*as they are commonly seen forming a lek close to the females to attract the female. A similar behavior was also observed in neotropical sand flies by Morrison et al. [41], where males of *Lu. longipalpis* arrived at animal pens two hours earlier than females. The other important difference between the two sexes of *Ph. orientalis* in the present study is that the activity of males was minimal between 05:00–06:00 h, however females were active during these periods. This may indicate that females exhibit both nocturnal and crepuscular behaviors, whereas males are clearly nocturnal in the area.

Determination of hourly parity rate of *Ph. orientalis* in the highland area indicated that the majority (75.5%) of the parous population were active before midnight mainly between 19:00–23:00 h. Similarly, Gebre-Michael & Lane [5] collected more parous females of *Ph. martini* and *Ph. celiae* between 20:00–22:00 h in southern Ethiopia. The abundance of this epidemiologically dangerous group in the early part of the night might be a risk factor for inhabitants working on the farms in the evening, or for people sleeping late outside their houses without using any protective measures, such as insecticide treated bed net. In contrast to the present observation, Roberts [40] in Oman encountered more parous females of *Ph. alexanderi* and *Se. clydei* in the later part of the night. In addition to determination of hourly parous rates, the activity of nulliparous females was also observed. These newly emerged females had two activity peaks throughout the night; the first between 22:00–23:00 h and the second between 03:00–04:00 h. The former coincides with the peak activity of males and this might be explained by mating behavior of the species. Such activity of nulliparous females of *Ph. orientalis* was also observed by Gebresilassie et al. [26]. A similar attempt was made in the lowland population of *Ph. orientalis* however; due to unfavorable weather condition enough number of sand flieswas not dissected to elucidate the hourly parous rates.

## Conclusions

The present study showed that *Ph. orientalis* populations in the highland and lowland areas have different activity patterns, with a peak in the early part of the night in the highland and in the latter part of the night in the lowland. Monthly variations in nocturnal periodicities were observed in both areas. Furthermore, the activity of the sand flies appears to be governed by temperature in the highland and relative humidity in the lowland, although the role of other factors cannot be ruled out. As the risk of acquiring *L. donovani* infections vary in the two areas, appropriate control strategies should be developed according to the activity of *Ph. orientalis* in the respective areas.

## Abbreviations

CDC: Centers for Disease Control and Prevention
CL: Cutaneous leishmaniasis
VL: Visceral leishmaniasis
